# Different roles of the RAAS affect bone metabolism in patients with primary aldosteronism, Gitelman syndrome and Bartter syndrome

**DOI:** 10.1186/s12902-022-00955-2

**Published:** 2022-02-11

**Authors:** Wangna Tang, Yun Chai, Hongwei Jia, Baoping Wang, Tong Liu, Hao Wang, Chenlin Dai

**Affiliations:** Endocrinology and Metabolism Disease Department, Tianjin Medical University General Hospital, 154# Anshan Road, Heping District, Tianjin, 300052 China

**Keywords:** Gitelman syndrome, Bartter syndrome, RAAS, Bone mineral density

## Abstract

**Background:**

Components of the RAAS may influence bone metabolism. Different roles of the RAAS are found in patients with primary aldosteronism (PA), Gitelman syndrome (GS) and Bartter syndrome (BS). We collected inpatient medical records including 20 patients with Gitelman syndrome (GS group), 17 patients with Bartter syndrome (BS group) and 20 age-matched patients with primary aldosteronism (PA group). We found the following results. (1) PA patients had significantly higher serum magnesium, potassium, plasma aldosterone, serum parathyroid hormone, urinary calcium and BMI (*p*<0.05) while significantly lower serum calcium and phosphorus (*P* < 0.05) than GS and BS patients. (2) Total hip and femoral neck bone mineral density (BMD) in PA patients were significantly lower than those in GS and BS patients (*P*<0.05). (3) GS patients had lower serum magnesium and urinary calcium than BS patients (*P* < 0.05). (4) Compared with BS patients, the vertebral BMD in GS patients were significantly higher (*P* < 0.05). So we believe higher aldosterone and PTH levels may be the reason that PA patients have lower hip BMD. Lower urinary calcium and inactivation of the NCC gene (Na-Cl cotransporter) in GS patients may have protective effects on vertebral bone mineral density.

**Conclusions:**

With persistence disordered RAAS, PA patients have lower BMD, especially hip BMD as compared with GS and BS patients. We presumed the lower renin and higher aldosterone level may be the reason. With the same level of renin and aldosterone, BS patients have lower vertebrate BMD than GS patients. Decreased urinary calcium excretion may be the reason.

## Introduction

This study is based on a female patient treated by us recently who had hyperparathyroidism caused by parathyroid cancer. She developed severe osteoporosis with multi-bone fractures. We measured her renin and aldosterone levels since she had hypo-potassium and normal blood pressure. Surprisingly, renin was more than 500 (normal 2.8–39.9uIU/ml) and aldosterone was also significantly increased. The blood potassium, renin and aldosterone of the patient returned to normal 1 week after the operation without other interventions. We were impressed by this strong interaction between PTH and RAAS. We wonder what PTH level and bone mineral density would be in patients with persistent disordered RAAS?

Primary aldosteronism (PA), Bartter syndrome (BS) and Gitelman syndrome (GS) are three endocrine diseases with disordered RAAS. Primary aldosteronism (PA) as primary hyperaldosteronism or Conn’s syndrome, is characterized by hypertension and hypokalemia, while Bartter syndrome (BS) and Gitelman syndrome (GS) as secondary hyperaldosteronism, characterized by hypokalemia, normal to low blood pressure, and metabolic alkalosis. Hypomagnesemia is less frequent than hypercalciuria in BS, while hypomagnesemia is an essential feature of GS, accompanied by hypocalciuria [1]. The RAAS is disordered in PA, BS and GS; however, the pathogenesis is not the same. Elevated aldosterone in PA is from the proliferation of the adrenal gland or adrenal adenoma, and the increased aldosterone in turn inhibits the secretion of renin. Renal tubular ion exchange channel mutations are found in GS and BS patients, and this may induce the proliferation of juxtaglomerular cells and the elevation of renin secretion, followed by increased aldosterone secretion [2, 3].

The interaction between the components of RAAS and PTH is well reviewed in the literature. It was found that plasma aldosterone level and ARR ratio were positively associated with plasma PTH level [4, 5]. High plasma PTH level can inactivate the vitamin D receptor (VDR) and increase the expression of renin, which can also directly affect the synthesis and release of aldosterone in plasma [6, 7]. It has also been reported that a high level of aldosterone may have a negative effect on bone [8–11]. However, there are few studies on whether the elevated aldosterone by different mechanisms may have the same effects on bone as PA, and whether this effect is mediated by PTH or other pathway. Therefore, we review the clinical data of Bartter and Gitelman syndrome patients and hope to answer the question.

## Subjects and methods

### Study subjects

The study population consisted of 57 patients, 30 men aged 15–55 years old and 27 women aged 15–66 years old. All patients enrolled in this study were inpatients who underwent detailed medical history, the general symptoms, signs and laboratory examination (including blood, urine, stool routine, biochemical tests, thyroid function, adrenocortical function, 24 h urine free cortisol, serum sex hormone, 24 h urine VMA, growth hormone and IGF-1). Patients who had a past or current disease that affects bone metabolism (such as pregnancy or lactation, nutritional diseases and gastrointestinal diseases, blood disease, chronic renal failure, low phosphorus, hyperthyroidism, Cushing’s syndrome, gonad hypofunction, hyperprolactinemia, diabetes, acromegaly and family hereditary metabolic bone disease, etc.) or who were taking drugs that affect bone metabolism (Such as glucocorticoids, heparin, anticonvulsants, immunosuppressants) were excluded. Exclusion criteria also include deficiency of potassium intake, vomiting, chronic use of diuretics or laxatives, and other causes of tubular dysfunction.

In all patients, aldosterone-renin ratio (ARR) screening tests were performed after adequate pharmacological elution of all interfering antihypertensive agents for at least 4 weeks.

Twenty patients were diagnosed with GS [4] according to the following criteria: 1) decreased serum potassium below the 3.5 mmol/L and because of the renal reasons; 2) decreased serum magnesium below the 0.7 mmol/L; 3) decreased urinary calcium; 4) metabolic alkalosis; 5) increased serum renin; 6) Low or normal-low blood pressure (< 140/90 mmHg); 7) normal kidney ultrasound performance; 8) combined with SLC12A3 gene mutation.

Seventeen patients with BS [5] according to the following criteria: 1) decreased serum potassium below the 3.5 mmol/L and because of the renal reasons; 2) decreased serum magnesium below the 0.7 mmol/L;3) normal or increased urinary calcium; 4) metabolic alkalosis; 5) increased serum renin; 6) Low or normal-low blood pressure (< 140/90 mmHg); 7) normal kidney ultrasound performance; 8) combined with CLCNKB gene mutation.

**Fig. 1:**
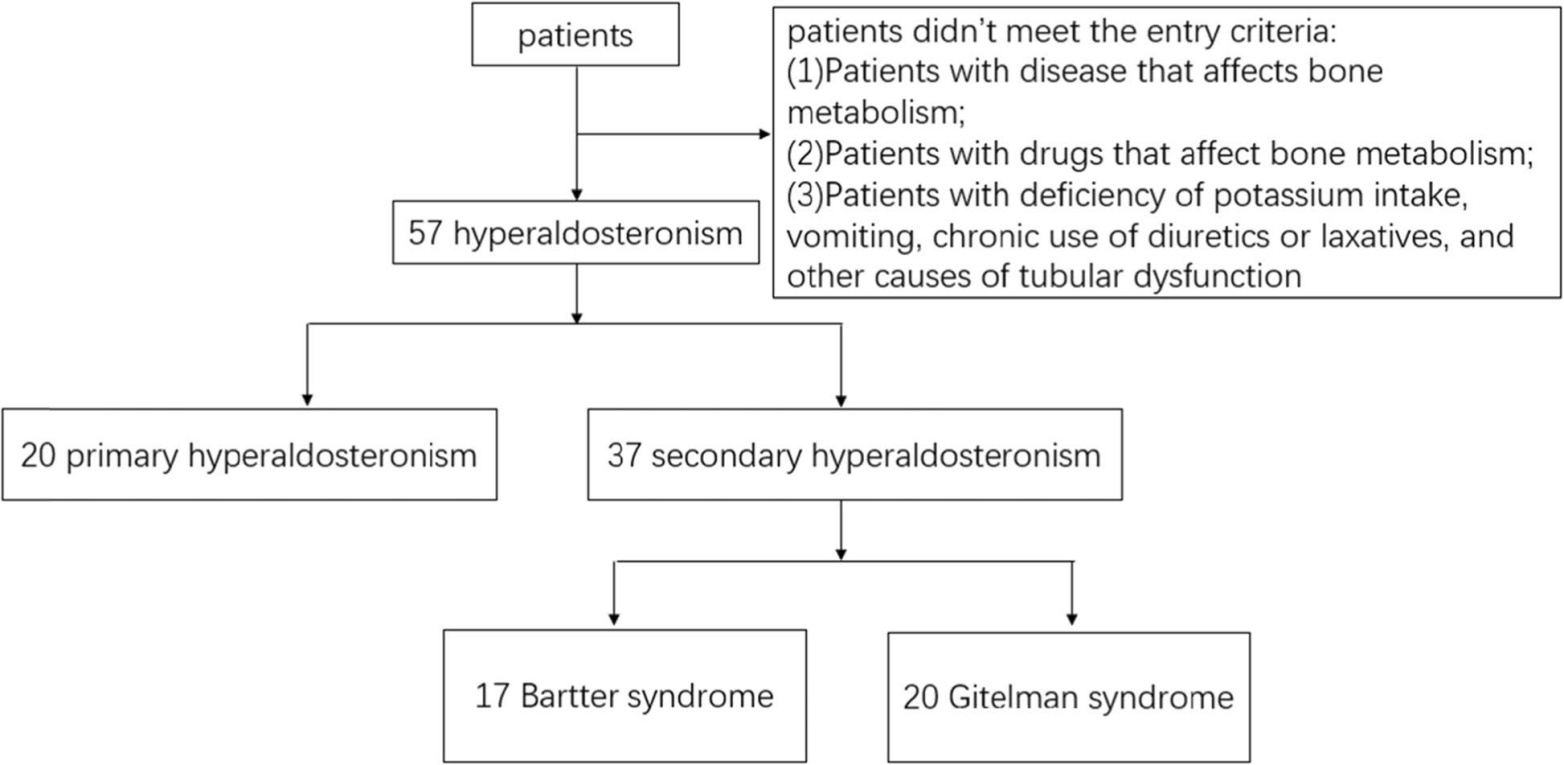
A diagram depicting the experimental design of this study

Twenty age-matched PA [12] patients according to the following criteria: 1) mild-to-moderate essential hypertension above 140/90 mmHg and resistant to three or more conventional antihypertensive drugs; 2) decreased serum potassium below the 3.5 mmol/L; 3) increased aldosterone /renin ratio (ARR) above the upper limit of normal range of 30; 4) one of the four tests (oral sodium loading, saline infusion, fludrocortisone suppression, and captopril challenge) is used in diagnosis; 5) adrenal computed tomography(CT) or adrenal venous sampling (AVS) are used in diagnosis as necessary. There is a diagram depicting the experimental design of this study for easier understanding (shown in Fig. 1).

All subjects were hospitalized patients first treated in Tianjin Medical University General Hospital between 2010 and 2019. The study was approved by the Institutional Review Board (IRB)/Ethical Committee of Tianjin Medical University General Hospital. And the ethics approval code was Ethical NO. IRB2015-YX-009.

## Methods

Blood and 24-h urine electrolytes, kidney and liver function, alkaline phosphatase, parathyroid hormone, 25-OHD, blood gas analysis, plasma renin activity (PRA) and aldosterone (Ald) were collected from patients in the supine position. Total calcium was corrected for serum albumin (Caalb-adj) according to the formula: Caalb-adj = total calcium + [(4-albumin expressed as g/dL) × 0.8]. PTH and 25OHD were detected by electrochemiluminescene assay. All the tests were done in the center laboratory of the hospital. BMD was measured using the GE Lunar Prodigy bone density scanner. All patients underwent abdominal ultrasonography. Adrenal lesions were confirmed by CT. (results in Tables 1 and 2).

**Table 1:** Comparison of clinical data between patients with primary aldosteronism and Bartter and Gitelman syndrome

Variable	PA group (20 cases)	Gits+Bartter group (37 cases)	*P*
Sex (male/female)	11/9	19/18	0.792(χ2)
Age, year	38.8 ± 10.1	36.1 ± 14.6	0.467
BMI, kg/m^2^	24.47 ± 4.19	21.79 ± 4.55	0.033
Albumin, g/L	42.70 ± 3.13	42.29 ± 3.20	0.650
Albumin-corrected serum calcium, mmol/L	2.23 ± 0.11	2.34 ± 0.19	0.019
Serum phosphate, mmol/L	1.09 ± 0.19	1.24 ± 0.21	< 0.01
Serum potassium, mmol/L	3.34 ± 0.54	3.02 ± 0.41	0.017
Serum creatinine, umol/L	61.60 ± 16.32	60.64 ± 16.75	0.837
Serum magnesium, mmol/L	0.88 ± 0.09	0.68 ± 0.17	< 0.01
Serum PTH, pmol/L	11.21 ± 7.10	3.62 ± 3.09	< 0.01
Serum 25-OHVD, nmol/L	36.20 ± 18.57	43.98 ± 21.80	0.183
Serum AKP, U/L	60.5 (55.25, 69.75)	60 (49.5, 68)	0.587
Serum renin, uIU/mL	1.50(0.60, 2.40)	5.4 (2.71, 34.55)	< 0.01
Serum aldosterone, ng/dL	27.00 (16.90, 38.35)	16.9 (12.25, 21.65)	< 0.01
Urinary calcium, mmol/24 h	5.62 (4.24, 7.76)	0.65 (0.46, 1.39)	< 0.01
L1-L4, BMD, g/cm^2^	1.115 ± 0.153	1.156 ± 0.158	0.351
Femoral neck, BMD, g/cm^2^	0.872 ± 0.141	0.971 ± 0.154	0.022
Total hip, BMD, g/cm^2^	0.926 ± 0.148	1.033 ± 0.157	0.016

**Table 2:** Comparison of clinical data between patients with Gitelman syndrome and Bartter syndrome

Variable	Gits group (20 cases)	Bartter group (17 cases)	*P*
Sex (male/female)	11/9	8/9	0.630 (χ^2^)
Age, y	36.3 ± 15.5	36 ± 13.9	0.951
BMI, kg/m^2^	21.99 ± 4.57	21.54 ± 4.66	0.770
Serum potassium, mmol/L	2.96 ± 0.31	3.10 ± 0.50	0.345
Albumin, g/L	41.70 ± 3.19	43.00 ± 2.76	0.224
Albumin-corrected serum calcium, mmol/L	2.34 ± 0.17	2.34 ± 0.23	1.000
Serum phosphate, mmol/L	1.29 ± 0.20	1.19 ± 0.21	0.193
Serum creatinine, umol/L	57.7 ± 16.81	64.11 ± 16.49	0.251
Serum magnesium, mmol/L	0.62 ± 0.16	0.74 ± 0.16	0.025
Serum PTH, pmol/L	3.58 ± 3.13	3.67 ± 3.13	0.932
Serum 25-OHVD, nmol/L	44.55 ± 18.88	43.30 ± 25.40	0.864
Serum AKP, U/L	61.00 (52.25, 67.50)	58.00 (46.50, 71.50)	0.636 (Non-parametric)
Urinary calcium, mmol/24 h	0.53 (0.27, 0.94)	1.36 (0.61, 3.70)	< 0.01 (Non-parametric)
Urinary magnesium, mmol/24 h	4.37 ± 1.43	3.92 ± 2.01	0.432
Urinary potassium, mmol/24 h	94.73 ± 52.27	83.82 ± 47.15	0.513
Blood gas PH, (7.35–7.45)	7.47 ± 0.031	7.47 ± 0.033	0.814
Serum renin, uIU/mL	4.53 (1.97, 73.52)	5.48 (3.09, 18.26)	0.962 (Non-parametric)
Serum aldosterone, ng/dL	17.35 (12.83, 19.65)	15.42 (11.60, 24.10)	0.924 (Non-parametric)
L1-L4, BMD, g/cm^2^	1.209 ± 0.170	1.095 ± 0.120	0.028
Femoral neck, BMD, g/cm^2^	1.008 ± 0.148	0.927 ± 0.155	0.114
Total hip, BMD, g/cm^2^	1.057 ± 0.160	1.003 ± 0.153	0.303

The measurement data with a non-normal distribution were expressed by M (P25, P75), others were expressed as ^−^x ± s

### Groups

Patients were divided into three groups: the primary aldosteronism group (20 cases), Gitelman syndrome group (20 cases) and Bartter syndrome group (17 cases). First, data from PA patients (20 cases) were compared with data from both patients with GS and BS (37 cases). Then, the data of GS patients were compared with those BS patients. The results are shown in Tables 1 and 2, respectively.

### Statistical methods

SPSS 25.0 statistical software was used for data analysis. The results are presented as the mean and standard deviation unless otherwise specified. The analysis of the count data was performed by χ2 test, and the comparison of the two pairs was performed by the independent sample t-test. The measurement data with a non-normal distribution were expressed by M (P25, P75), and the nonparametric test was used for comparison between groups. *P* < 0.05 was considered statistically significant.

## Results

Compared with PA patients, BS and GS patients had higher serum calcium and phosphorus levels, lower BMI, and lower serum potassium, magnesium, PTH, plasma aldosterone and urine calcium levels (*P* < 0.005). PA patients had lower renin levels and lower femoral neck and total hip bone mineral densities than BS and GS patients (P < 0.005). The results are shown in Table 1.

Serum magnesium and urinary calcium were lower in GS patients than in BS patients (*P* < 0.05). GS patients had higher lumbar spine bone mineral density than BS patients (*P* < 0.05). The results are shown in Table 2.

## Discussion

The RAAS is disordered in primary hyperaldosteronism (PA), Bartter syndrome (BS) and Gitelman syndrome (GS); however, the pathogenesis is not the same. PA patients have higher aldosterone and lower renin levels while GS and BS patients have higher renin and higher aldosterone levels. Renin was reported to have an independent positive effect on bone [13]. So we compared PA patients with GS and BS patients.

Our clinical data showed that plasma aldosterone, urinary calcium, and serum parathyroid hormone in PA patients were significantly higher and serum calcium and renin levels were significantly lower than those in GS and BS patients. We believe that the increased aldosterone may be the root cause except renin. Now, it is believed that aldosterone may have a direct effect on the parathyroid gland as well as on bone because the mineralocorticoid receptor was found in the parathyroid tissue and on bone cells [14, 15]. At the same time, increased aldosterone may promote the excretion of urinary calcium, leading to a decrease in serum calcium, which in turn stimulates the secretion of parathyroid hormone [16, 17]. Elevated parathyroid hormone in patients with primary aldosteronism may affect bone health, especially cortical bone, as there is a significant decrease in hip bone mineral density. However, the vertebral BMD is similar when compared with BS and GS patients. Magnesium may influence the synthesis of parathyroid hormone. There is a significant decrease in serum magnesium in BS and GS patients. This may be the other possible reason that PA patients have higher PTH levels compared with BS and GS patients.

Serum magnesium and urinary calcium are significantly lower in GS patients than in BS patients. However, the PTH and aldosterone levels were similar in both groups. Therefore, we believe that it is the increased aldosterone, not the decreased magnesium, that induces higher PTH levels in PA compared with GS and BS.

When comparing the data of GS patients and BS patients, although the numbers in each group were relatively low, we found that there was a significant difference in the lumbar vertebrate bone mineral density between these two groups and no significant difference in the hip bone mineral density. Except for urinary calcium and serum magnesium, other indexes of bone metabolism, such as vitamin D, parathyroid hormone, phosphorus and calcium, were similar in both groups. The specific pathogenesis of the reduction in urinary calcium in GS patients is not yet clear. Laurence Nicolet-Barousse and others found that in humans, loss of NCC gene function leads to GS [18]. The NCC gene edits (gene locus SLC12A3) Na-Cl cotransporter. The thiazide-sensitive Na-Cl cotransporter is located in the apical membrane of the distal epithelial cells of the renal distal convoluted tubule and is responsible for sodium reabsorption [19]. Inactivated Na-Cl cotransporter in mice represents an experimental model of GS [20]. Ncc−/− mice have a similar phenotype to GS patients. Increased bone mineral density in Ncc−/− mice as well as in GS patients was observed, indicating that inactivation of the NCC gene leads to an increase in bone mineral density. The NCC gene may be a new determinant factor of bone quality [18].

Therefore, patients with primary aldosteronism who take mineralocorticosteroid receptor antagonists should be followed up not only for blood pressure and serum potassium but also for bone mineral density, especially hip BMD. Currently, only symptomatic treatment can be given to patients with Gitelman and Bartter syndrome. Due to the relatively early onset of the disease, long-term follow-up is needed to evaluate the influence of the long-lasting disease, especially for BMD of patients with Bartter syndrome.

### Limitations

Bartter and Gitelman syndrome are rare diseases which are diagnosed at young age. Patients with such diseases are seldom to evaluate their bone mineral density. That is why the number of the patients included in this study is small. So the limited sample size may affect the validity of the study results.

## Conclusion

With persistence disordered RAAS, PA patients have lower BMD, especially hip BMD as compared with GS and BS patients. We presumed the lower renin and higher aldosterone level may be the reason. With the same level of renin and aldosterone, BS patients have lower vertebrate BMD than GS patients. Decreased urinary calcium excretion in GS group may be the reason.

Feedback and negative feedback mechanisms in the endocrine system are complex, the circulating levels of any hormone may not be sufficient to explain the clinical results. Moreover, BS and GS are rare diseases, so larger sample sizes and prospective studies are needed to corroborate our experimental conclusions.

## Data Availability

The datasets generated and/or analysed during the current study are available in the the Baidu Netdisk repository, as this website record: https://pan.baidu.com/s/1PpWgy9rKDtNBJ0XbKeid3w, with the access code 1018.

## Abbreviations

PA: Primary aldosteronism
GS: Gitelman syndrome
BS: Bartter syndrome
BMD: Bone mineral density
Na-Cl cotransporter: NCC
VDR: Vitamin D receptor
ARR: Aldosterone-renin ratio
PRA: Plasma renin activity
Ald: Aldosterone
MRA: Mineralocorticoid receptor antagonism
